# Short- and Intermediate-Term Outcomes of Preterm Infants Receiving Positive Pressure Ventilation in the Delivery Room

**DOI:** 10.1155/2013/715915

**Published:** 2013-01-17

**Authors:** Megan O'Reilly, Po-Yin Cheung, Khalid Aziz, Georg M. Schmölzer

**Affiliations:** ^1^Department of Pediatrics, University of Alberta, Edmonton, AB, Canada T6G 2R3; ^2^Division of Neonatology, Department of Pediatrics, Medical University Graz, 8036 Graz, Austria; ^3^Department of Newborn Medicine, Royal Alexandra Hospital, 10240 Kingsway Avenue NW, Edmonton, AB, Canada T5H 3V9

## Abstract

Although recent advances in neonatal care have improved survival rates, rates of bronchopulmonary dysplasia remain unchanged. Although neonatologists are increasingly applying gentle ventilation strategies in the neonatal intensive care unit, the same emphasis has not been applied immediately after birth. A lung-protective strategy should start with the first breath to help in the establishment of functional residual capacity, facilitate gas exchange, and reduce volutrauma and atelectotrauma. This paper will discuss techniques and equipment during breathing assistance in the delivery room.

## 1. Introduction

Approximately 20% of premature infants require breathing support at birth [1, 2]. An international consensus on resuscitation suggests equipment and techniques if infants fail to initiate breathing [3]. It is agreed that positive pressure ventilation (PPV) is the cornerstone of respiratory support at birth [3]. During the application of PPV in the delivery room (DR) the lungs of preterm infants are exposed to potentially injurious tidal volumes (*V*_*T*_) [4, 5]. Although neonatologists are familiar with the concept of reducing lung injury and are increasingly careful in the neonatal intensive care unit (NICU) to apply PPV strategies that are gentle to the lung, the same gentle approach has not been translated into practice in the DR [6]. Ideally, a lung-protective strategy should start immediately after birth. At birth, the lungs of very preterm infants are uniquely susceptible to injury because they are structurally immature, surfactant-deficient, fluid-filled, not supported by a stiff chest wall, and are unable to generate adequate end expiratory pressure to maintain open alveoli [6]. To facilitate early development of functional residual capacity (FRC), reduce atelecto- and volutrauma, and improve oxygenation, sustained inflations (SIs), positive end expiratory pressure (PEEP), and continuous positive airway pressure (CPAP) have been advocated [7–15].

This paper summarizes the various methods available to clinicians for the provision of positive pressure ventilation to preterm infants in the DR, the impact on clinical outcomes, and potential areas for further research.

## 2. Search Strategy

The aim of this article was to review the available literature about delivery room interventions and their effect on outcomes in newborn infants. We reviewed books, resuscitation manuals and articles from 1960 to present with the search terms “Infant, Newborn,” “Delivery Room,” “Neonatal Resuscitation,” “Intubation,” “Surfactant,” “Positive Pressure Respiration,” and “Continuous Positive Airway Pressure.”

## 3. Respiratory Support in the Delivery Room

The purpose of PPV is to establish FRC, deliver an adequate *V*_*T*_ to facilitate gas exchange, and stimulate breathing while minimizing lung injury [6]. The International Liaison Committee on Resuscitation and various national resuscitation guidelines recommend equipment and techniques for neonatal resuscitation [16–18].

### 3.1. Ventilation Devices during Respiratory Support in the Delivery Room

There is currently limited evidence to guide clinicians' choice of device for providing PPV in the DR [19]. Self-inflating bags, flow-inflating bags, or T-piece devices may all be used for mask ventilation. A self-inflating bag, however, does not provide PEEP or CPAP [6, 20]. An attached PEEP-valve provides inconsistent PEEP and cannot deliver CPAP [21–24]. A flow-inflating bag provides variable- and operator-dependent PEEP [16, 25]. With a T-piece device a more consistent, predetermined level of PEEP and PIP can be delivered [5, 21, 22]. In addition, a T-piece device has been shown to be the most accurate device for delivering a sustained inflation breath [17, 22, 26, 27]. 

### 3.2. Respiratory Function Monitor

**Figure 1:**
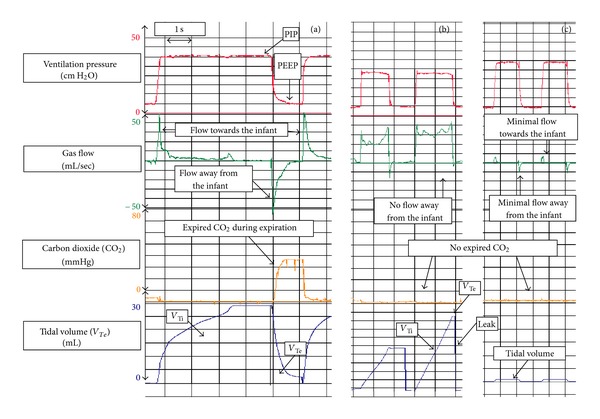
The figure shows how an RFM can help to optimize PPV in a 26-week preterm infant with 800 gram birth weight. In (a) during inflations the airway pressure increased form baseline (PEEP) to the set PIP. Similar gas flow towards and away from the infant indicates no leak around the mask. In addition the *V*_*T*_ wave returns to baseline indicating good mask ventilation. Expired CO_2_ can be observed once the *V*_*T*_ wave returns to baseline. With the start of the next inflation expired CO_2_ drops to zero. In (b) PEEP and PIP are achieved; however gas flow only moves towards the infant and only minimal gas flow away from the infant indicating mask leak. The *V*_*T*_ wave shows inspiratory *V*_*T*_ (*V*_Ti_) but no expiratory *V*_*T*_ (*V*_Te_). Mask leak indicated as a straight line in the *V*_*T*_ curve, and no expired CO_2_ displayed. In (c) displays airway obstruction which can be identified by minimal or no gas flow movements, no expired CO_2_, and no or minimal *V*_*T*_ waves.

The use of respiratory function monitor (RFM) has been described during neonatal simulation [28], neonatal resuscitation [29, 30], and neonatal transport [31]. A Respiratory Function Monitor uses a small, low dead space flow sensor (~1 mL), which is placed between a ventilation device and a facemask or endotracheal tube [30]. The monitor can be set to continuously display airway pressure, gas flow, and tidal volume waves. It also measures and displays numerical values for peak airway pressure, PEEP, CPAP, *V*_*T*_, respiratory rate, and expiratory minute ventilation [30]. Adverse events (e.g., mask leak or airway obstruction) can be identified by observing the displayed waveforms [30]. The leak between mask and face or around an endotracheal tube is expressed as a percentage of the inspired *V*_*T*_. Leak is graphically presented as the difference in area under the flow curves above (inflation) and below (deflation) zero flow (Figure 1(b)) [30]. Several observational studies in the DR have reported on the advantages and disadvantages of an RFM during neonatal resuscitation. Recently, a randomized trial by Schmölzer et al. compared the additional use of an RFM with clinical assessment versus clinical assessment alone and reported significant reduction in mask leak, significant increase in CPAP use, and significant less intubation in the DR [29]. Although this is promising, further trials are warrant.

### 3.3. Mask Ventilation in the Delivery Room

Using airway maneuvers (e.g., jaw thrust or chin lift) to maintain airway patency is a crucial step during mask ventilation in adults and children [32]. However in newborn and infants several factors can reduce the effectiveness of mask ventilation, including poor face mask technique resulting in leak or airway obstruction, spontaneous movements of the baby, movements by or distraction of the resuscitator, and procedures such as changing the wraps or fitting a hat [33, 34]. Delivery room studies have shown that mask leak and airway obstruction are common problems during PPV [5, 33, 34]. Both leak and obstruction are usually unrecognized unless expired CO_2_ detectors or RFM (Figure 1) is used [33, 34].

### 3.4. Assessment of Mask Ventilation

If infants fail to initiate spontaneous breathing immediately after birth, PPV should be given [16]. A rapid increase in heart rate is the most important clinical sign for adequate mask ventilation [16, 35, 36]. If no heart rate increase is observed, chest wall movements should be assessed to gauge mask ventilation [16]. However, the current neonatal resuscitation guidelines do not describe how chest wall movement should be assessed [16]. Two observational studies in the DR compared clinical assessment with measurements of an RFM [4, 5]. Schmölzer et al. compared chest rise with *V*_*T*_ measurement during mask PPV in the DR [5]. Assessing chest wall movement during mask PPV whilst standing at the infant's head was difficult and unreliable [5]. However, limitations of this study were the inexperience of the resuscitators and the potential obstructed view of the resuscitators by the ventilation device [5]. Poulton et al. compared chest rise observed from two different angles (head view versus side view) and different level of experience (junior staff versus senior staff) [4]. Overall the accuracy of clinical assessment of chest wall movement was poor and did not appear to be influenced by either the observers' position or the level of experience. However, more resuscitators were unable to assess chest wall movements while performing PPV than those observing from the side [4]. These two studies demonstrate that resuscitators were unable to accurately assess chest wall movements during mask PPV. The additional use of an RFM to continuously measure and displays *V*_*T*_ delivery might improve the effectiveness of neonatal resuscitation. During mask PPV an RFM continuously display *V*_*T*_ wave forms which can be used to guide mask ventilation. The clinical team can identify mask leak or airway obstruction as well as high or low *V*_*T*_ delivery to guide ventilation. A recent randomized trial by Schmölzer et al. demonstrated that an RFM additional to clinical assessment demonstrated significant reduction in leak during mask PPV in preterm infants in the DR [29]. 

### 3.5. Mask Leak

Mannequin studies demonstrated large mask leaks during simulated mask ventilation, and operators were usually unaware of the extent of mask leak [37, 38]. Observational studies in the DR reported similar results with mask leak exceeding 75% in 50% of analyzed resuscitations [5, 34]. The leak between the mask and the face is an enemy of mask PPV, causing a reduction in tidal volume delivery and impairing resuscitation efforts. A mannequin study demonstrated that operators observing RFM graphics (Figure 1) were able to reduce mask leak during PPV [39]. A recent randomized controlled trial comparing mask PPV in the DR performed with either an RFM visible or masked showed similar results [29]. Observation of flow waves significantly reduced mask leak from 54% to 37% [29]. Furthermore, fewer infants were intubated or required oxygen at five minutes after birth, and more infants left the DR on CPAP [29]. Although no difference in any long-term outcomes was observed, the results of this study may indicate that flow wave guidance improves mask PPV and decreases short-term adverse outcomes [29].

### 3.6. Airway Obstruction

Current resuscitation manuals suggest that during mask PPV airway obstruction may be due to (i) manual compression of the soft tissues of the neck and tongue, (ii) hyperextension or flexion of the head, or (iii) the face mask being held on the face so tightly that it obstructs the mouth and nose [16, 33, 34].

Two observational studies in the DR reported on airway obstruction during resuscitation of preterm infants [33, 34]. Finer et al. used a colorimetric CO_2_ detector to identify obstruction during mask PPV. They found airway obstruction in 75% of infants receiving PPV in the DR [33]. Although CO_2_ detectors can be very useful to assess effective ventilation, they do not differentiate between an inadequate *V*_*T*_, airway obstruction, or circulatory failure [40–43]. In contrast an RFM, which displays flow and tidal volume signals (Figure 1), may distinguish mask leak and airway obstruction, [30, 34]. A recent observational study in the DR showed that severe airway obstruction defined as a reduction in *V*_*T*_ of >75% occurs in 25% of infants receiving mask ventilation [34]. 

Several airway maneuvers, such as jaw thrust or chin lift, are recommended in children and adults to maintain airway patency during resuscitation [16]. An airway obstruction has been reported in 50% of cases when either chin lift or jaw thrust was applied during mask PPV, while using the combination of both, no airway obstruction was observed [32]. Similar studies are needed in newborn infants to clarify the best head and airway position.

### 3.7. Tidal Volume Delivery

During PPV a peak inflation pressure (PIP) is chosen with the assumption that this will deliver an adequate *V*_*T*_ [5]. However, the delivered *V*_*T*_ is rarely measured, and therefore airway pressure is not adjusted accordingly [4, 5]. A low *V*_*T*_ may be insufficient to achieve adequate gas exchange and may cause hypercapnia and atelectotrauma, whereas excessive *V*_*T*_ may cause hypocapnia and volutrauma [6]. Both low and excessive *V*_*T*_ delivery promote release of inflammatory mediators, which contribute to bronchopulmonary dysplasia (BPD) [44, 45]. In addition, clinicians struggle to achieve a balance between aerating the distal gas exchange units (alveoli) without overdistending the lung causing damage [6]. An animal study demonstrated that a few large manual inflations can damage the lungs [46]. Tidal volumes similar to this study have been reported during mask PPV of preterm infants [4, 5]. In addition, several animal studies demonstrated that PPV with high *V*_*T*_s contributes to lung injury [45, 47]. Using a lung simulator Kattwinkel et al. demonstrated that operator adjusted to compliance changes faster when *V*_*T*_ was displayed compared to pressure [48, 49]. A recent randomized control trial compared *V*_*T*_ guidance with clinical assessment during mask PPV in the DR in infants <32 weeks of gestation [29]. Mask leak was significantly decreased in the RFM visible group; however *V*_*T*_ was similar in both groups [29]. Promisingly, the infants in the RFM visible group received less high *V*_*T*_ (>8 mL/kg) delivery compared to the masked group [29], which have been shown to contribute to lung injury [45, 50]. 

### 3.8. Sustained Inflation

In preterm infants a lung protective strategy should be started at birth to support lung fluid clearance and to establish FRC. Establishment of lung inflation in apneic newborn infants can be achieved with either shorter or longer inflation times [16]. In an experimental non-breathing rabbit model of neonatal resuscitation, a prolonged sustained inflation (SI) of 20 s coupled with PEEP resulted in a rapid increase in FRC as did PEEP alone when compared to PPV with or without PEEP [51]. Evidence in preterm infants comes from observational and randomized studies [7–9, 52]. Lindner et al. introduced a series of interventions in the DR that included giving a 15 s SI [8]. They observed a dramatic reduction in DR intubation rate from 84% to 40%, and the proportion of preterm infants never intubated during their admission at their institution increased from 7% to 25% [8]. Similarly, Lista et al. compared an initial 15-second SI in addition to PPV to control infants who did not receive SI [7]. They reported a reduction in surfactant (45% versus 61%) mechanical ventilation (51% versus 75%) and postnatal steroid (10% versus 25%) use. In addition, infants surviving without BPD increased from 7% to 25%, and mean duration of mechanical ventilation (5 versus 11 days) and oxygen therapy (21 versus 31 days) among infants <29 weeks was reduced [7]. Lindner et al. randomized 61 infants <29 weeks to receive a 15 second SI and PPV or PPV alone through a single nasal prong in the DR [8]. Although no difference in mortality, severe intraventricular hemorrhage, or BPD was observed, 30% to 40% of preterm infants were not intubated or mechanically ventilated within the first 48 hours after birth [8]. Harling et al. randomized 52 preterm infants to an initial 5-second SI and PPV compared to PPV alone and did not find a difference in cytokines measured in bronchoalveolar lavage fluid [52]. Te Pas and Walther randomized 207 preterm infants <33 weeks to an initial 10-second SI followed by nasal CPAP compared to mask PPV without PEEP [9]. Lower intubation rates in the DR (17% versus 36%), shorter duration of ventilatory support, and BPD (22% versus 34%) were observed in the infants randomized to SI/CPAP compared to those receiving mask PPV without PEEP [9]. Although these studies suggest that SI has the potential to reduce BPD, the results have to be interpreted with caution. Cohort studies are subject to confounders and can at best suggest an association between the use of an SI and improved outcomes. For example, infants in Te Pas and Walther's study were on average 500 g heavier compared to those in Lindner et al.'s study [8, 9]. Both Lindner et al.'s and Te Pas and Walther's studies reported more than one DR care change, with SI being just one element among [8, 9]. In addition, the randomized studies were not adequately powered to detect differences in important clinical outcomes [8, 9, 52]. Consequently, it is not possible to determine how many, if any, of the differences observed between the groups were related to the use of SI. Large randomized controlled studies of SI in preterm infants are urgently needed.

### 3.9. Continuous Positive Airway Pressure or Intubation

Observational studies have reported an association between decreased rates of BPD and increased use of early CPAP [53–56]. Avery et al. compared BPD rates in eight NICUs with one center having a significant lower BPD rate with much greater use of CPAP compared to the other centers [53]. Van Marter et al. reported that rates of BPD differed substantially between Columbia and Boston centers (4% versus 22%) [54]. Initial respiratory management was more likely to include mechanical ventilation (75% versus 29%) and surfactant (10% versus 45%) at Boston centers compared to Columbia, respectively [54]. A retrospective analysis of 261 preterm infants compared intubation and ventilation at birth with CPAP and reported lower mortality and rates of surfactant administration, BPD, or intraventricular hemorrhage in infants receiving CPAP [55]. Surprisingly, patent ductus arteriosus was more common among infants receiving CPAP [55]. Two randomized trials compared PEEP/CPAP with no PEEP in the DR [15, 21]. In a feasibility study Finer et al. randomized 104 extremely low birth weight infants to receive CPAP/PEEP or no CPAP/PEEP in the DR. The aim of the study was to sue CPAP/PEEP to avoid routine endotracheal intubation and to explore the CPAP or PEEP in the DR. Although no differences in rates of intubation, death, and BPD were reported, the use of CPAP/PEEP as initial respiratory management was feasible [15]. Dawson et al. randomized infants <29 weeks' gestation and reported no difference in oxygen saturation or heart rate at 5 min, mortality, rate of intubation, or BPD [21]. Two large trials randomized 1926 infants between 24 and 29 weeks of gestation to receive CPAP or endotracheal intubation at birth [12, 13]. The COIN trial reported fewer days of ventilation and reduction of surfactant use in infants receiving CPAP than those in infants endotracheally intubated at birth [13]. Worryingly, infants in the CPAP group had a significantly higher incidence of pneumothorax [13]. The SUPPORT trial randomized 1316 infants to receive CPAP or intubation and surfactant. Infants in the CPAP group had lower rates of postnatal steroids and had fewer days of mechanical ventilation than those in the latter group. However mortality and BPD rates were similar between groups in both trials [12]. Nonetheless, the results suggest that respiratory support in the DR should be started with CPAP before intubation and surfactant are considered.

### 3.10. Surfactant Administration

Surfactant deficiency is a contributing factor in the development of respiratory distress syndrome (RDS) and has become the standard of care for the treatment of RDS. Systematic reviews from randomized trials 15 years ago showed that prophylactic surfactant administration reduced mortality and initial inspired oxygen requirement for intubated infants <30 weeks' gestation or with birth weight less than 1250 g [57]. This led many to advocate routine intubation and surfactant administration for infants at risk of RDS [58–60]. However, the care of very immature babies has changed considerably over the last decade, and early CPAP has become an accepted alternative to endotracheal intubation and surfactant treatment [12, 13, 61–65]. In a retrospective cohort study, selective intubation of ELBW infants resulted in a significantly reduced need for intubation, lower incidence of BPD, intraventricular hemorrhage, and decreased length of hospital stay as compared to routine intubation [8]. A recent Cochrane review summarized that early stabilization on CPAP with selective surfactant administration compared to prophylactic surfactant administration and mechanical ventilation lowers the risk of BPD or death [66]. Verder et al. described his “INSURE” technique “Intubation-Surfactant-Extubation” which aimed to intubate infants only for surfactant delivery while on nasal CPAP [61]. In a multicenter randomized trial the INSURE technique reduces the need for mechanical ventilation; however no difference in important long-term outcomes (e.g., BPD) was reported [61]. The major criticism of the INSURE technique was the necessity of analgesia and naloxone to reverse the potential respiratory depression because of opioids. Various techniques of minimally invasive surfactant therapy have been described. Kribs et al. described surfactant delivery in spontaneous breathing infants on CPAP [63]. Using a flexible feeding tube positioned in the trachea with Magill's forceps surfactant is delivered [63]. Compared to historical controls the rates of mortality, severe intraventricular hemorrhage, and pulmonary interstitial emphysema were significantly reduced [63]. Two further observational cohort studies by Kribs et al. showed similar results [64, 65]. In both studies the rates of mechanical ventilation, BPD, and death were significantly lower compared to infants receiving intubation and mechanical ventilation [64, 65]. A recent multicenter randomized control trial using the Kribs technique reported a decrease in need for mechanical ventilation in the group who received surfactant while one CPAP [67]. However, no differences in mortality, BPD or other serious adverse events were observed. Alternative Dargaville et al. described “The Hobart Method” were surfactant is in stilled using a 16 gauge vascular catheter. With this technique the catheter is inserted through the vocal cords and surfactant instilled [68]. The catheter is then immediately withdrawn and CPAP reinstituted. A recent observational study by Dargaville et al. reported a reduction in need for intubation <72 h in infants receiving minimally invasive surfactant therapy compared with controls [69]. Although infants receiving minimally invasive surfactant therapy had shorter duration of oxygen therapy, duration of ventilation and incidence of BPD were similar [69]. Currently a large RCT using “The Hobart Method” is underway.

## 4. Conclusion

Ideally, a lung-protective strategy should start immediately after birth. At birth, the lungs of very preterm infants are uniquely susceptible to injury because they are structurally immature, surfactant-deficient, fluid-filled, and not supported by a stiff chest wall. To facilitate early development of functional residual capacity, reduce atelecto- and volutrauma, and improve oxygenation, various methods have been advocated. However, randomized control trials are urgently needed to investigate short- and intermediate-term outcomes.

## Abbreviations

PPV: Positive pressure ventilation
*V*_*T*_*n*: Tidal volumes
NICU: Neonatal intensive care unit
DR: Delivery room
FRC: Functional residual capacity
SI: Sustained inflation
PEEP: Positive end expiratory pressure
CPAP: Continuous positive airway pressure
RFM: Respiratory function monitor
PIP: Peak inflation pressure
BPD: Bronchopulmonary dysplasia
RDS: Respiratory distress syndrome.
